# Adaptability of different mechanisms and kinetic study of methane combustion in steam diluted environments

**DOI:** 10.1038/s41598-022-08648-5

**Published:** 2022-03-17

**Authors:** Subhankar Mohapatra, Mani Bhusan Rajguru Mohapatro, Amjad A. Pasha, Radi A. Alsulami, S. K. Dash, V. Mahendra Reddy

**Affiliations:** 1 Department of Mechanical Engineering, Indian Institute of Technology Kharagpur, Kharagpur, West Bengal 721302 India; 2 Aerospace Engineering Department, King Abdulaziz University, Jeddah, 21589 Saudi Arabia; 3 Department of Mechanical Engineering, King Abdulaziz University, Jeddah, 21589 Saudi Arabia

**Keywords:** Energy science and technology, Engineering

## Abstract

The chemical kinetics of methane oxidation in a steam-diluted environment are studied in the present study. Various well-validated mechanisms for methane combustion are adopted and compared with experimental data. Ignition delay, laminar flame speed, and emissions for CH_4_ combustion with steam dilution are discussed. Cumulative relative error parameter was determined for all mechanisms considered in this study to evaluate the prediction level in quantifiable terms. Reaction pathways under no and steam-diluted environments are analyzed, and key elementary reactions and species are identified in these conditions. The analysis gives a relative idea of the applicability of some of the reduced mechanisms for the diluted steam conditions. This study aims to guide future computational fluid dynamics simulations to accurately predict combustion characteristics in these conditions. Computations of laminar flame speed from GRI-3.0, Aramco3.0, Curran, and San Diego mechanisms were the most precise under diluted steam conditions. Similarly, for the calculation of ignition delay of methane under the steam dilution, the Aramco mechanism and the Curran’s mechanism were able to predict the experimentally observed values most closely. Sensitivity study for the OH concentrations shows that the H-abstraction of methane from OH radicals has an opposing trend with dilution for Aramco and GRI-3.0 mechanism. On the other hand, CO and NO emissions were reduced significantly, with the dilution increased from 0 to 20%. The third-body effect of steam is observed to dominate the deviation observed between the detailed and reduced mechanism. For low operating pressure conditions, the GRI-3.0 mechanism gives an excellent prediction, whereas, for applications like gas turbines and furnaces, Aramco-3.0 and Curran mechanisms can be adopted to give good results. The San Diego mechanism can be chosen for low computational facility purposes as it shows very good predictions for ignition delay and laminar flame speed computations.

## Introduction

The drastic increase in pollution has forced the need to identify new technologies and improve the old methods to reduce emissions during combustion. Different methods adopted by researchers to lower the peak temperature of the combustion to control the NO_x_ emissions include fuel/air staging^1–3^, Lean premixed combustion^4–6^, Exhaust or Flue gas recirculation (EGR/FGR), MILD combustion^7–13^, emulsion fuel technique^14–16^. Dilution in the combustion zone is another method to obtain a more homogeneous combustion regime where the reaction temperature is reduced^17^.

Some common diluents include recirculating the product gases like CO_2_ and N_2_ and inert gases like Ar and He. Being readily available and cheap, steam has been gaining greater interest as a prevalent diluent amongst researchers. The primary advantage of steam addition is that it alters combustion chemistry by actively participating in the elementary reactions and primarily changes the concentration of reactive radicals like H and OH. Steam addition also modifies the physical properties like temperature, which can change the course of the reaction pathway. Combustion systems are often studied numerically, as it is not possible to conduct experiments for all conditions each time because of the time and cost constraints. The computational studies, primarily CFD, require importing the mechanism files to model the chemistry. However, these mechanisms were developed for a limiting range of parameters like pressure, temperature, dilution levels, etc. Each mechanism is not suitable for all ranges of operating conditions and needss to be compared with the experimental results for its applicability. Recent studies^18,19^ show the requirement for modifications of existing mechanism files to predict the combustion chemistry at different mixture compositions. Thus, the numerical schemes should accurately capture the chemical and thermal effect of steam dilution to allow further analysis using steam dilution conditions. This warrants the need to identify the mechanisms and combustion characteristics that prevail in the diluted environment. The present study aims to provide a comparative study of the performance of some of the well-validated mechanisms in capturing the combustion chemistry in steam dilution conditions and to understand the differences between the mechanisms. These mechanisms were investigated against the comprehensive experimental dataset from the literature. The combustion characteristics in these conditions are also presented.

The addition of water/steam in the combustion system reduces the peak temperature because of its high specific heat capacity, which helps in holding thermal heat without increasing the temperature. Thermal NO_x_ is dependent on the peak temperature, thus reducing with steam/water addition to either fuel or air^20–27^. The addition of steam alters the NO_x_ formation path, which can also help in the reduction of the NO_x_ emission^23–25,28^. Steam dilution also increases the OH radicals,, which helps oxidize CO–CO_2_^29,30^. Lyu et al.,^28^ varied the steam dilution in H_2_/Air system from 0 to 30% while varying the equivalence ratio from 0.9 to 3 and pressure up to 3 atm. They observed a constant reduction of laminar flame speed with pressure beyond steam dilution of 12%. However, the flame speed below the 12% dilution level increases to 2.5 atm pressure and then reduces monotonously. These fluctuations are attributed to the direct reaction and the third-body effect from the steam dilution^31^. The discrepancies observed between the experimental and the numerical results were thought to be the amplified uncertainties of the rate constants of the elementary reactions at the elevated pressure and steam diluted environments. In a previous study by Koroll and Mulpuru^32^, the reduction of laminar flame speed for H_2_/O_2_ mixture with dilution has been established that was due to the high third-body efficiency of the elementary reaction H + O_2_ + M ⇔ HO_2_ + M, which is an exothermic reaction. The increase in temperature due to the rise in the rate of this exothermic reaction results in a thinner flame and high flame speed. They also observed that the increase in the third-body efficiency increased the concentration of OH radicals in the system.

The impact of steam on the kinetics has been widely studied for H_2_ fuel. These studies acts as a guide towards understanding the H_2_–O_2_ chemistry and NO_x_ emissions in the steam diluted environments. However, hydrocarbon fuels being the most conventional fuel in present scenarios, needs to be extensively investigated under these conditions. The most simple and smallest fuel amongst all hydrocarbon fuels is methane. Thus, a better way to study steam addition on hydrocarbons is by adopting methane as the fuel. Methane is also one of the most extensively studied fuels whose kinetics is well discussed in the literature. Few studies have been conducted to analyze methane combustion with steam dilution^29,33–35^. These studies indicate that the chemical effect and the physical effect of H_2_O addition have a significant influence on the combustion characteristics and emissions. Li et al.^36^ added N_2_, CO_2_, Ar, and H_2_O(v) as diluents up to 10% in oxidizer for CH_4_/air combustion. They found steam to be the most effective than CO_2_, N_2,_ and Ar in reducing NO_x_ emission.

Shareh et al.^33^ observed that the role of the chemical effect of steam dilution increases with an increase in the temperature, whereas the physical effect reduces with the temperature. Similarly, in the experimental work of Mazas et al.^37^, a significant chemical effect of the steam dilution on the laminar flame speed of CH_4_/Air flames is observed and attributed this effect due to the high chaperon efficiency of H_2_O in the third-body reactions. However, this chemical effect is almost negligible with an increase in oxygen. Gurentsov et al.^38^ studied ignition delay in the multicomponent hydrocarbon-air mixtures for hypersonic aircraft applications with different cases of steam, argon and helium added to the mixtures. They observed good prediction between the experimental and numerical results, computed with GRI-3.0 and Dautov and Starik model^39^, for steam addition cases. However, they reported a significant reduction (about 2–3 times) in ignition delay with steam dilution from the experiments, which couldn’t be captured using the computations. Boushaki et al.,^40^ demonstrated the effect of steam addition (in terms of specific humidity) on the laminar flame speed of methane combustion both experimentally and numerically at atmospheric pressure conditions. They considered the GRI-3.0 mechanism for the computations and achieved a good level of prediction.

Jach et al.^41^ have compared 15 different mechanisms for their predictions for ignition delay times of C2–C6 alkenes and acetylene with shock tubes results only. They calculated Pearson linear correlation coefficients for the detailed mechanisms to assess comparable similar behavior that could be ensured from the hierarchical nature of mechanism generation. Baigmohammadi et al.^42^ have also considered a wide range of experimental data set of equivalence ratios (0.5–2), initial temperatures (800–2000 K), pressures (1–80 bar), and dilution (N_2_ and Ar) levels (75–95%) for estimating ignition delay of C1–C2 hydrocarbons (namely methane, ethane, and ethylene). They have adopted the C3-NUIG mechanism for computing ignition delay and have compared the results with their experimental results and the data available in the literature. In another study, Hu et al.^43^ have developed a reduced skeletal mechanism with 22 species for CH_4_/O_2_ combustion after comparing seven detailed mechanisms in high CO_2_ concentration conditions. They observed that USC-II mechanism was able to predict better as compared to the other mechanisms and was considered for the reduction. The reduced mechanism was able to predict within 10% of the detailed mechanism. A recent study by Zhang et al.^44^ encapsulates the prediction from most of the well-validated mechanisms for a wide range of experimental estimations of ignition delay times using rapid compression machine (RCM) and shock tubes for methane combustion. They observed that most mechanisms could predict the ignition delay measured using shock tubes more satisfactorily than the ignition delay measured with RCMs. They have also compared mechanisms for methane combustion under diluted environments that primarily include N_2_ and Ar and feebly on more reactive radicals like CO_2_ and H_2_O. However, the details on the effect of diluents were not discussed in their study. These previous studies show that the mechanisms for C1–C4 hydrocarbons are compared primarily for ignition delay parameters. The laminar flame speed, being an important characteristic, also need to be compared with the available mechanisms in the literature. Olm et al.^45^ have considered both laminar flame speed and ignition delay parameters to compare different mechanisms for H_2_ combustion systems.

Moreover, under the influence of steam dilution and the use of different rate and thermodynamic parameters observed in the well-validated mechanisms, the kinetics may vary from the conventional knowledge^28,44^. Steam as diluent is unique to change the combustion chemistry by dissociating to reactive radicals. In addition, researchers are using the reduced skeletal mechanisms in their CFD studies to analyze the combustion phenomenon for a wide range of applications. Although the reduced mechanisms in the literature are well-validated with their detailed parent mechanism and experimental values and predict results within an acceptable range of all combustion parameters, these validation studies aren’t carried for high dilution cases. Therefore, it becomes necessary to analyze the adaptability of the reduced mechanisms and to show whether it validates well as their parent mechanisms at the high dilution conditions. Recently, Hu et al.^13^ observed a different trend for NO_x_ with equivalence ratio in MILD combustion regime for methane combustion as opposed to the conventional knowledge of variation of NO_x_ emission with equivalence ratio from their experimental and chemical kinetics study. They observed important routes including NNH and prompt NO, N_2_O intermediate and NO-reburning which dictates total NO_x_ formation at for different operating temperature and equivalence ratio (0.5–1) range. The combustion of CH_4_/air and CH_4_/O_2_ also ahs different characteristics since high CO2 concentrations is observed for the oxy-fuel combustion which also effects the combustion chemistry inside the domain^43^. Thus, it becomes imperative that the kinetics for specific applications are well understood. However, it can be noted that the experimental data set for the diluted conditions are not available in rich numbers, unlike pure hydrocarbon fuels (without dilution). Therefore, the comparison of different mechanisms could only be carried out with a few well-validated studies.

The present study attempts to identify the suitable mechanism for steam diluted combustion with methane fuel. Various well-validated detailed and reduced chemical mechanisms have been developed which are used to analyze methane combustion^46–54^. The relative comparison is conducted among all the mechanisms by considering the laminar flame speed and ignition delay times, the crucial parameters in combustion characteristics. Nine mechanisms, including seven detailed and two reduced mechanisms, are considered in the analysis. Ignition delay for CH_4_/air and CH_4_/Ar/O_2_ are estimated using a closed homogeneous reactor (CHR) model. The ignition delay parameters are compared with the experimental work of Donohoe et al.^29^. The flame speed model available in CHEMKIN is adopted to compare the laminar flame speed computed from various kinetic mechanisms with the available experimental results^34,37^. The effect of steam dilution on the reaction pathways is presented for a detailed and reduced mechanism to understand the effect of the number of species on the oxidation pathways for no and steam diluted conditions. Important chemical reactions and kinetics enhanced with the adoption of steam dilution are understood from the sensitivity studies shown in this paper. The effect of steam addition on emissions, namely CO and NO, is presented, and the key reactions controlling their net production rates are discussed.

## Numerical modelling

The effect of steam dilution on methane oxidation is shown from the available experimental work and numerical simulations carried out using thewell-validated mechanisms at the same operating conditions. The present investigation involving the detailed mechanisms and the reduced mechanisms is provided in Table 1. The detailed mechanisms include GRI-3.0, Aramco 3.0, USC-II, San Diego, Curran et al., and Glarborg et al. The reduced mechanisms include DRM 22 and USC II's recently developed reduced mechanism. Similarly, the Fundamental Fuel Chemistry Model (FFCM-1) mechanism was developed primarily for C0–C2 fuels. All mechanisms are well-validated for a wide range of temperature equivalence ratio and pressure applications and have been adopted in the literature. However, the different sets of reactions and rate parameters in these well-validated mechanisms may predict the combustion characteristics differently for any operating condition.

**Table 1:** Different mechanisms adopted in the present study.

Mechanisms	No. of species	No. of reactions	Reference
GRI-Mech 3.0	53	325	^46^
Aramaco 3.0	589	3037	^47^
USC II	110	784	^48^
Curran et al.,	113	710	^49^
San Diego	58	270	^50^
DRM 22	21	84	^51^
Glarborg et al.	154	1397	^52^
USC reduced	50	373	^53^
FFCM_1	38	291	^54^

To assess these effects, a wide range of validation studies with experimental results are required. In the present study, chemical kinetic analysis is conducted to understand the impact of dilution on the flame characteristics with different mechanisms. Ignition delay, laminar flame speed, and emissions are crucial parameters for comparing the different mechanisms. The experimental results of Donohoe et al.^29^ and Gurentsov et al.^38^ are considered to study and compare the effect of steam dilution on the ignition time of methane. Ignition delay calculations were carried out using 0-D closed homogeneous reactor model. A constant volume model was adopted for all computations. For the cases of Donohoe et al.^29^ a parametric study with varying dilution levels (0–30%), temperature (1400–2200 K), equivalence ratio (0.5, 1, and 2), and pressure (1–30 atm) are carried out for the validation of ignition delay phenomenon with Ar/O_2_ used as the oxidizer. The oxidizer was highly diluted with an argon composition is 98% by volume. The default tolerances were adopted in the study. Similarly, Gruentsov et al. carried out experiments for a pressure range of 3.3–7.6 atm, temperature range of 1300-1885 K while the mixture was maintained for stoichiometric level. The mole fraction of steam addition was kept constant at 0.08333 for the steam dilution cases. The cases for steam dilution with N_2_ and Ar as the buffer gases in the reactant mixtures were only considered in this study. The addition of CO as the buffer gas in their study weren’t considered in the present analysis as CO also can affect the chemical and physical behavior of the combustion system. Ignition delay estimation was considered using the peak OH level, temperature inflection point and temperature change of 300 K for all mechanisms for the test case of stoichiometric mixture at atmospheric pressure condition. Very close estimations were observed between the above three criteria, with OH criteria being marginally close to the experimentally observed ignition delay times. Following this, the OH criteria was adopted to calculate the ignition delay for rest of the cases^29^.

Laminar flame speed calculation was carried out using the mechanisms mentioned in Table 1 for the experimental conditions of Mazas et al.^37^, Galmiche et al.^34^, and Boushaki et al.^40^ with the flame speed model. The unburned mixture temperature and equivalence ratio adopted for Mazas et al. cases are 373 K and 0.5–1.5 respectively for the CH_4_/O_2_/N_2_/H_2_O mixture at 1 atm pressure conditions. The oxygen concentration of 50% in the reactant mixture was considered in this study. Galmiche et al.^34^, on the other hand, haven’t varied the oxygen level while keeping the unburned mixture temperature at 393 K for the stoichiometric condition. H_2_O dilution of up to 25% was considered for this study. Boushaki et al.^40^ have added steam as relative humidity from 0 to 100% to the mixture at 300 K and 330 K, and 1 atm pressure. Soret effect caused due to thermal diffusion effect, is enabled in the simulations. Grid independent solution was obtained for each case by varying the gradient and curvature controls. It was observed that for different mechanisms, different values of the gradient and curvature controls were observed for the grid-independent solution, and it was generally found to vary between 0.01 and 0.03. Grid refinement is done until the change in the velocity is less than 0.1 cm/s. The maximum number of grid points was kept at 1000. The axial length of 10 cm was considered in this study to calculate flame speed. The default solver parameters of the flame speed model were considered for the computations.

The mechanisms mentioned in Table 1 are used to simulate the experimental results from the literature to compute the laminar flame speed and the ignition delay, respectively. In addition, a comparative study amongst the performance characteristics for different mechanisms was carried out by computing a cumulative relative error parameter, which measures the deviation of the simulated results from the experimental results. This is expressed as:

1 $$ Cumulative\, relative\, error, CRE\left( {\xi_{s} } \right) = \mathop \sum \limits_{n} \frac{{\left| {N_{i} - E_{i} } \right|}}{{E_{i} }} $$

2 $$ Cumulative\, relative \,error, CRE\left( {\xi_{\tau } } \right) = \mathop \sum \limits_{n} \frac{{\left| {{\text{ln}}\left( {N_{i} } \right) - {\text{ln}}\left( {E_{i} } \right)} \right|}}{{{\text{ln}}\left( {E_{i} } \right)}} $$

where *N*_*i*_ and *E*_*i*_ are the computed and experimental value of flame speed or ignition delay for the given operating condition of pressure and temperature, and *n* is the varying parameter for the given operating condition, i.e., temperature for ignition delay and equivalence ratio for laminar flame speed calculation. Equation (1) and (2) represents the cumulative relative errors for laminar flame speed calculations and ignition delay calculations, respectively. The choice of two different formulas for the CRE is due to the different nature of the scatter of the errors associated with the laminar flame speed with equivalence ratio and ignition delay times with temperature^43–45,55^. The ignition delay times vary in the logarithmic scale, whereas the laminar flame speed varies on the linear scale. The unit of the ignition time,$\tau$ extracted from the literature and the present computations is $\mu s$, and the same was used while estimating the errors. The summation operation considers the overall absolute error, which results in avoiding the error averaging. This CRE helps identify the mechanism that could best predict the experimental values. The sensitivity analysis and reaction pathway study is further carried out with this best-predicted mechanism to analyze the effect of steam dilution on the combustion characteristics. The net rate of production $(r_{ij}^{p} )$ and consumption $(r_{ij}^{c} )$ of *i*th species from the *j*th reaction are computed, as given in Eqs. (3) and (4), respectively.

3 $$ r_{ij}^{p} = \frac{{{\text{max}}\left( {\vartheta_{ij} , 0} \right)q_{j} }}{{\mathop \sum \nolimits_{j = 1}^{N} {\text{max}}\left( {\vartheta_{ij} , 0} \right)q_{j} }} $$

4 $$ r_{ij}^{c} = \frac{{{\text{min}}\left( {\vartheta_{ij} , 0} \right)q_{j} }}{{\mathop \sum \nolimits_{j = 1}^{N} {\text{min}}\left( {\vartheta_{ij} , 0} \right)q_{j} }} $$

where $\vartheta_{ij}$ and *q*_*j*_ are the stoichiometric coefficient and reaction rate, respectively of the *i*th species of the *j*th reaction. *N* represents the total number of elementary reactions in the mechanism considered. The net rate of production (ROP) and consumption of a particular species from the reaction helps in understanding the direction in which the reaction pathway is moving. Further, the important reactions and species obtained from the sensitivity analysis are normalized to a matrix of local sensitivity coefficient (*S*^*n*^). It is expressed as

5 $$ \begin{aligned} S^{n} = \left( {\frac{{k_{j} }}{{c_{i} }}\frac{{\partial c_{i} }}{{\partial k_{j} }}} \right) = \left( {\frac{{\partial \ln c_{i} }}{{\partial \ln k_{j} }}} \right) \\ \end{aligned} $$

where $k_{j}$ and $c_{i}$ are rate the constant of reaction *j*th and concentration of the *i*th species and $\frac{{\partial c_{i} }}{{\partial k_{j} }}$ signifies the sensitivity coefficient relative to the *i*th species for the *j*th reaction rate. The laminar flame speed model analyzes CO and NO emissions under the diluted steam conditions. The operating conditions and mixture compositions adopted to compute the cases of Galmiche et al. numerically are considered to show the variation of production rates of the radicals like H, OH and species like CO and NO along the one-dimensional length. The unburnt reaction temperature of 393 K and stoichiometric mixture condition is maintained for all computations to calculate the net rate of production of the radicals. Default solver settings with windward differencing are considered to obtain a grid-independent solution for no and diluted conditions. Reaction pathway for no and diluted environments at stoichiometric condition was further analyzed using a perfectly stirred reactor (PSR) model. The PSR model is a better way to understand the reaction kinetics behavior with a large recirculating flow pattern, which is observed in many practical applications. This model considers a steady-state gas energy equation solver with default solver settings. The reactor temperature and the pressure are set to 1300 K and atmospheric condition, respectively whereas the inlet temperature of the mixture is kept constant at 393 K. The residence time for the reactant mixture is kept constant at 0.5 s.

## Results and discussion

### Effect of steam dilution on ignition delay

**Figure 1:**
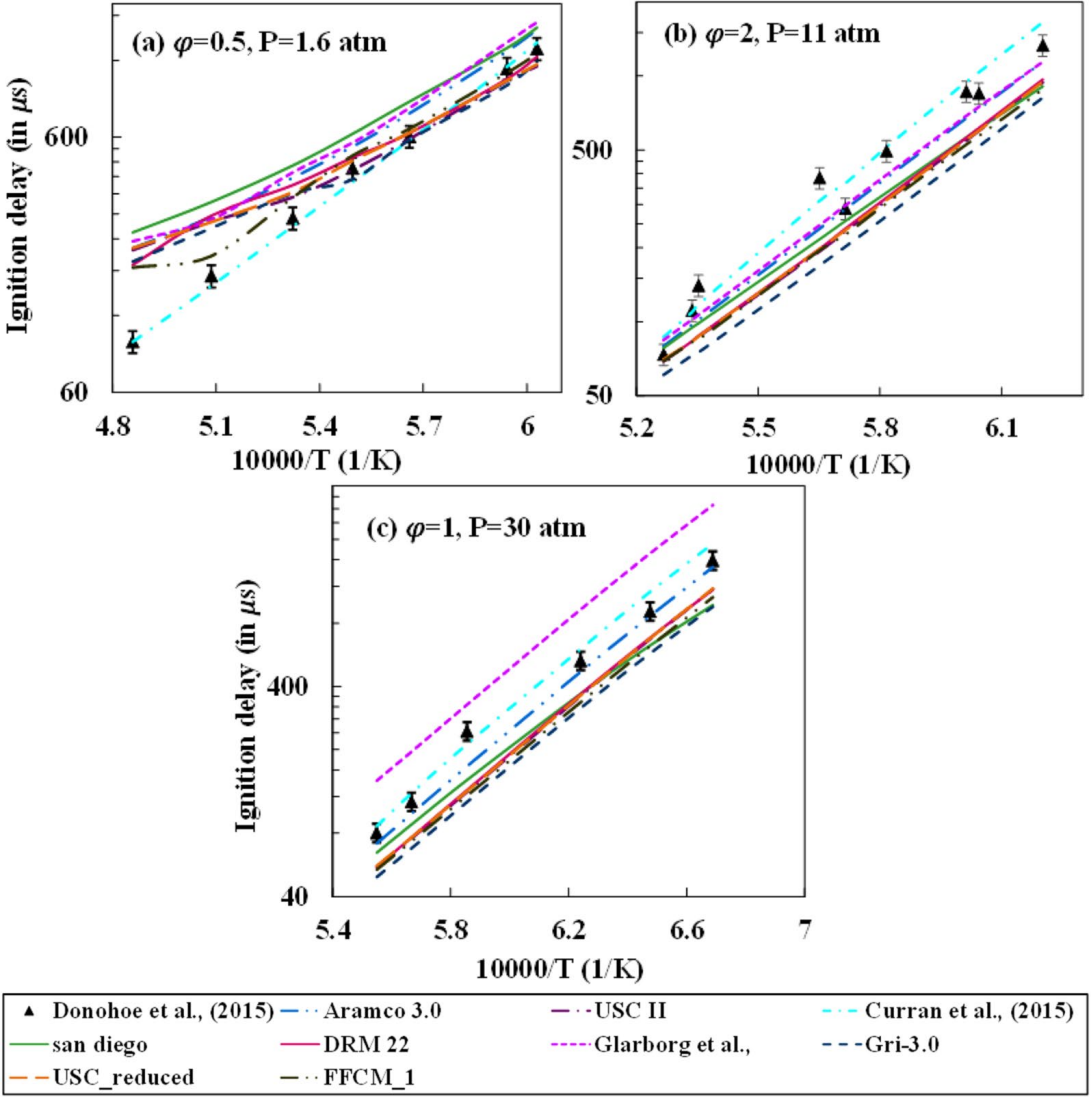
Comparison between different mechanisms for no steam dilution and CH_4_/Argon/O_2_ mixtures with 98% Argon by volume.

**Figure 2:**
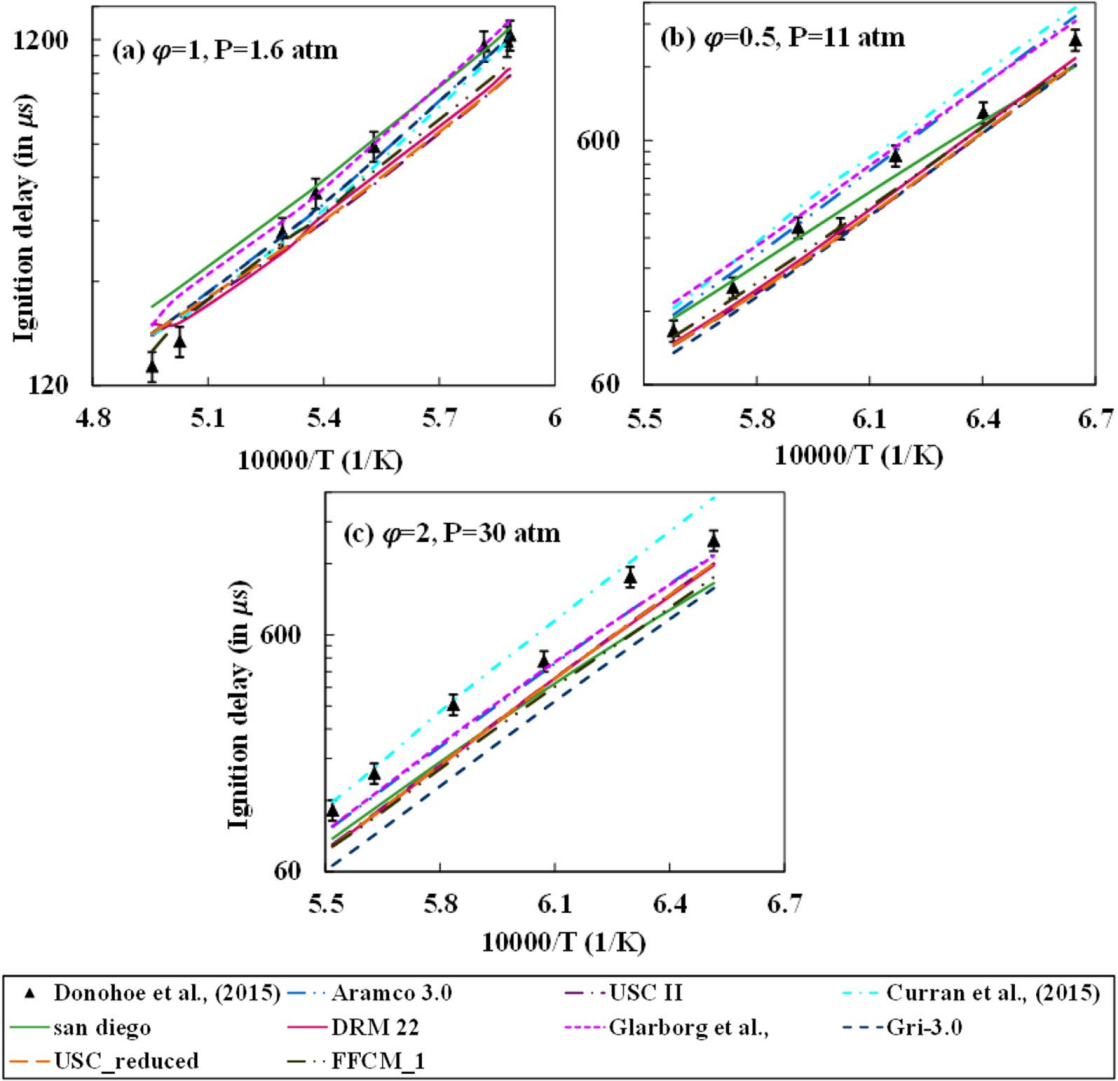
Comparison between different mechanisms for 10% steam dilution and CH_4_/Argon/O_2_ mixtures with 98% Argon by volume.

**Figure 3:**
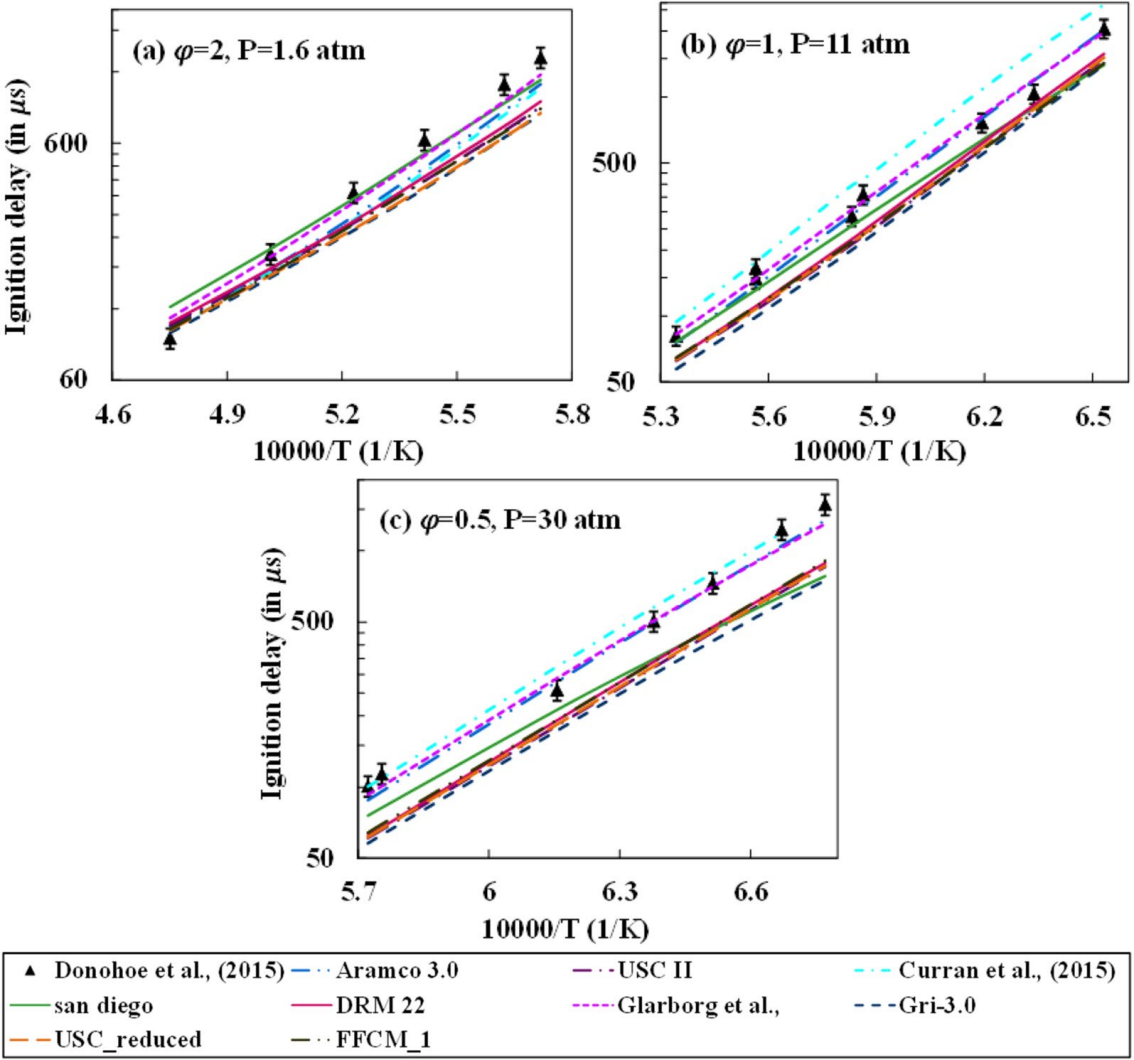
Comparison between different mechanisms for 30% steam dilution and CH_4_/Argon/O_2_ mixtures with 98% Argon by volume.

The ignition delay time is an essential parameter for describing the fuel oxidation characteristics and is an ideal indicator for flame stabilization. The experimental studies of Donohoe et al.^29^ and Gurentsov et al.^38^ are considered in the present study to compare the ignition delay prediction of methane with steam dilution with the different mechanisms as mentioned in Table 1. Donohoe et al. varied the equivalence ratio $(\varphi )$ (0.5, 1, and 2), pressure (1.6, 11, and 30 atm), and steam dilution level (0, 10, and 30%). The uncertainty in the experimental determination of the ignition delay values for their work was prescribed to be 10%. In their study, nine operating conditions were generated after applying an L9 Taguchi matrix, and the studies were carried out for these operating conditions. However, two parameters were varied simultaneously in any two cases out of the nine cases. Thus, the effect of any single parameter was not studied singularly in their investigations and thus can’t be compared with the present numerical simulations. The variation of ignition delay for three different steam dilution cases of no dilution, 10%, and 30% dilution is shown in Figs. 1, 2, and 3, respectively. The ignition delay reduces with pressure due to localization of the combustion zone and thereby promoting better collision efficiency. For the zero dilution case, all mechanisms observed a good prediction at higher pressure conditions, with Aramco and Curran et al.^49^ mechanism giving the best match. However, at a low low-pressure condition of 1.6 atm, the deviation from the experiments is observed to be very large at high-temperature conditions for all mechanisms except for the Curran et al.^49^ mechanism. With an increase in pressure, GRI 3.0 mechanism prediction reduces significantly. FFCM-1 mechanism over-predicts ignition delay for high temperature and under-predicts at low temperature at 1.6 atm pressure, whereas it constantly under-predicts ignition delay for all temperature conditions at high-pressure conditions.

Figure 2 shows 10% steam dilution conditions for the operating pressure levels and equivalence ratio. Unlike the 0% dilution case, a better prediction is observed for all mechanisms across pressure and equivalence ratio values. However, a similar behavior for the no dilution case is observed for the GRI 3.0 and DRM 22 (a reduced form of its previous version), where the prediction offsets with pressure. It is important to note that all mechanisms except Curran et al.^49^ under-predict the ignition delay at 30 atm with the prediction from the Glarborg mechanism predicting the closest to the experimental values. The under-prediction increases gradually with a rise in pressure for the San Diego mechanism. Figure 3 shows the ignition delay prediction for 30% steam dilution. The predictions from Aramco, Glarborg, and Curran mechanisms are better than the rest of the mechanisms at high dilution conditions. Aramco's mechanism starts to under-predict the ignition delay whereas the Curran mechanism over-predicts with a rise pressure. The under predictions for the GRI-mechanism increase with pressure, and the performance is least amongst the rest at 30 atm. The effect of steam addition has no significant effect on the prediction of the FFCM-1 mechanism as the qualitative and quantitative change in ignition delay estimation with varying temperature and pressure at 10% and 30% dilution cases are similar to no dilution case. The under-prediction of ignition delay increases with the increase in pressure.

**Figure 4:**
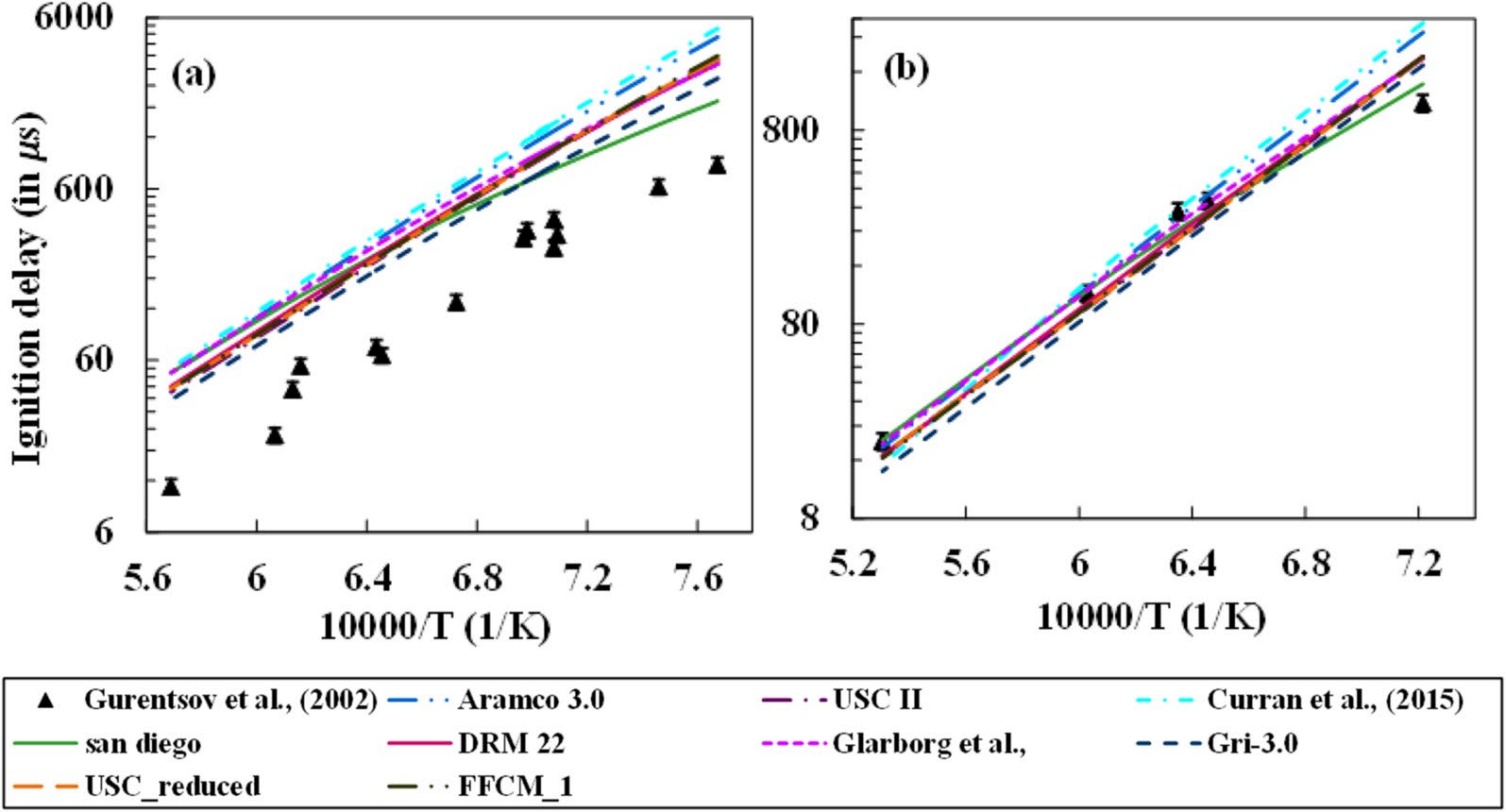
Comparison between different mechanisms for steam dilution with the buffer gases: (**a**) N_2_ and (**b**) Ar at different operating conditions^38^.

Figure 4 shows the numerical computations to evaluate the ignition delay times for the experimental conditions of Gurentsov et al. using the mechanisms considered in the present study. Figure 4a,b show the steam dilution cases with N_2_ and Ar as the buffer gases in the mixture, respectively. A large deviation in the predictions is observed from all mechanisms for N_2_ buffer gas, with San Diego and GRI-3.0 mechanisms showing a better match than the other mechanisms. However, Curran and Aramco-3.0 mechanisms, which performed very well for the cases of Donohoe et al.^29^, couldn’t capture the ignition delay in the hypersonic conditions. On the other hand, the overall agreement from all mechanisms improved significantly for the Ar buffer gas. The difference in behavior for the two buffer gases was assumed to be the different collision efficiency of the buffer gases in the third-body reactions of H_2_–O_2_ chemistry.

**Figure 5:**
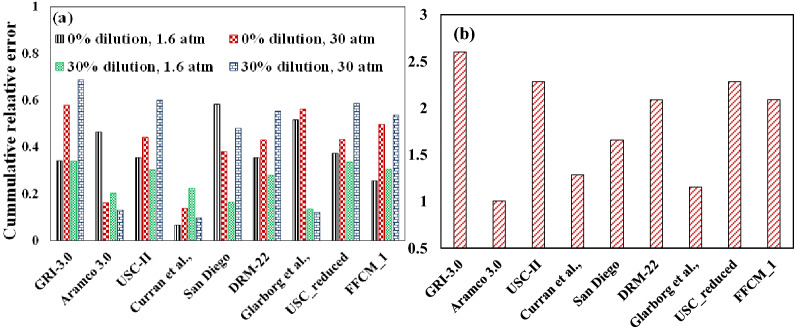
The cumulative relative error for different mechanisms for (**a**) cases with no and high dilution and 1 atm and 30 atm of Donohoe et al. and (**b**) combined dilution cases reported in the literature.

Although each mechanism considered in the present study matches the experimental values within an agreeable range (except the N_2_ addition cases for Gurentsov et al.,^38^), a significant variation is observed between the computed results from the mechanisms. As described in Sect. 2, cumulative relative error parameters estimate ignition delay prediction errors for different dilution and operating conditions are plotted and shown in Fig. 5. Figure 5a shows the extreme levels of pressure and dilution levels considered from the works of Donohoe et al.^29^, i.e., 1 and 3 atm for pressure and 0 and 30% steam dilution. This will help recognize the mechanisms showing the best efficiency at high pressure and diluted environments. Sensitivity analysis is then performed on the best-fit mechanism to identify the key reactions which affect ignition delay in the diluted environments. The best-fit mechanism is identified as the mechanism with the lowest CRE index computed for all dilution cases from the literature,^29,38^, as shown in Fig. 5b. Here, the effect of the equivalence ratio is neglected while calculating the mean deviation of the computed values from the experimental results.

Figure 5a shows that the GRI-3.0 mechanism performs reasonably well with increased dilution at low pressure compared to a high-pressure condition. The Aramco 3.0 mechanism, a more comprehensive mechanism, performs better than the rest at high pressure and dilution conditions. USC, DRM-22, San Diego, and USC_reduced mechanism behaves similarly at these conditions as the error reduces with pressure for no dilution and increases with pressure at 30% dilution. Glarborg model showed high error values at no dilution conditions while the deviation reduces significantly at 30% steam dilution conditions. FFCM-1 model behaved similarly for no and 30% dilution case at 1.6 atm, whereas the error increases slightly with 30% dilution at 30 atm pressure. The closest prediction was found to be with the Curran mechanism for the overall range. The Aramco 3.0 mechanism considerably improved its estimation with an increase in dilution and pressure, whereas the error values from GRI-3.0, USC-II, USC_reduced version, DRM-22, San Diego and Glarborg mechanism were on the higher side. Therefore a comparison between the mechanisms is necessary to understand the reason behind this deviation. This can be studied from the sensitivity analysis of these mechanisms for the desired parameter to find the reason behind these inconsistencies. In order to compare with the overall performance of the mechanisms in the diluted steam environments, the CRE index is plotted for all datasets of Donohoe et al.^29^ and Gurentsov et al.^38^ with dilution as shown in Fig. 5b. This shows that Aramco-3.0, Curran et al., and Glarborg et al. were able to estimate ignition delay values very close to the experimental data, whereas the GRI-3.0 mechanism showed a greater deviation from the experimental results. The reason behind the marginally poor prediction from the GRI-3.0 mechanism could be due to the high-pressure environments, irrespective of dilution levels, where the mechanism doesn’t perform much better. Thus, Aramco 3.0 and GRI-3.0 mechanisms were considered for the sensitivity studies as the Aramco mechanism was observed to perform better and GRI-3.0 mechanism has shown the most disagreement with the experimental results for the conditions adopted in this study.

**Figure 6:**
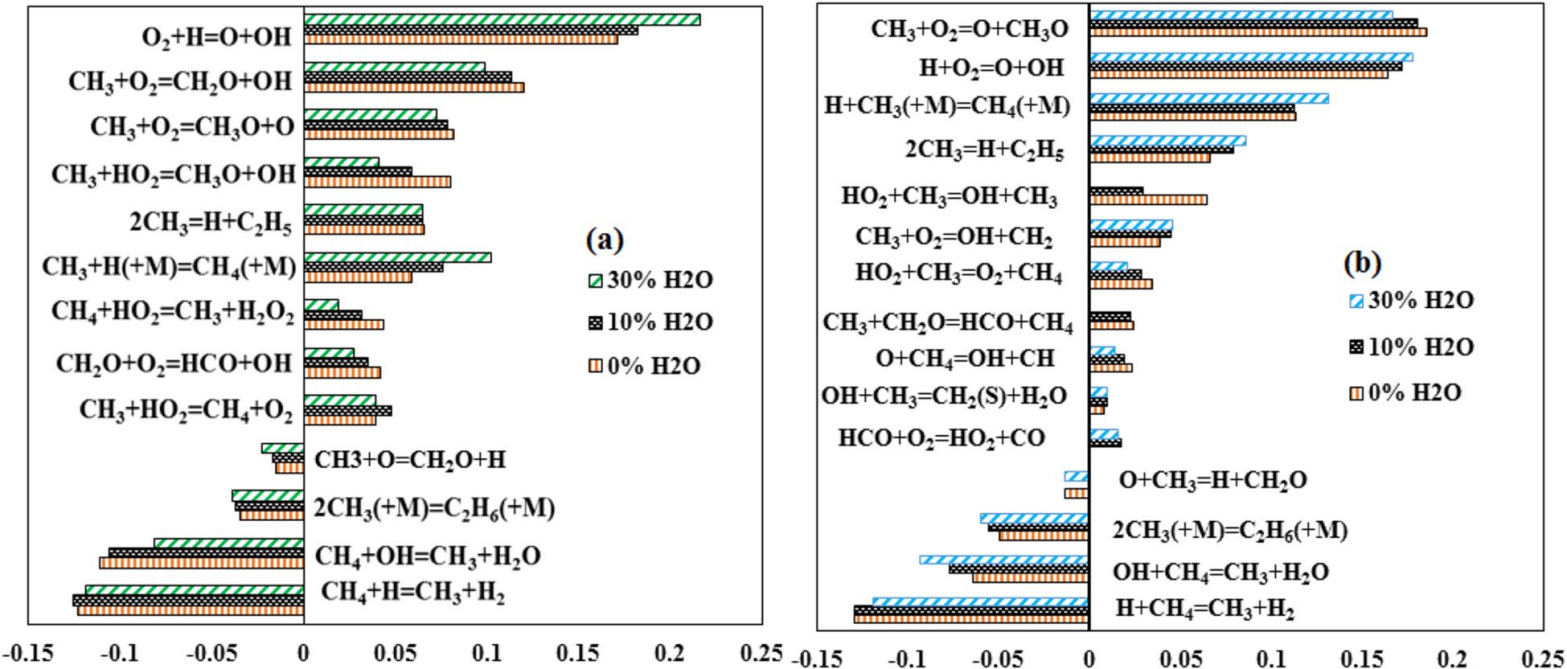
Sensitivity analysis of OH formation rate for different dilution cases using (**a**) Aramco and (**b**) GRI-3.0 mechanism for stoichiometric mixture at 1.6 atm and 1800 K.

Sensitivity analysis for OH formation rate is performed using the Aramco and GRI-3.0 mechanism, as shown in Fig. 6. OH is a very reactive radicals and helps the propagation of flame. Thus, it is chosen as the criteria to understand the behavior of the ignition time scale. The flux and sensitivity analysis is done at the time of 20% fuel (CH_4_) oxidation^49^. The sensitivity analysis is carried out for the stoichiometric mixture and 1.6 atm pressure. The steam is added to the premixed reactant mixture. A constant temperature of 1800 K is maintained for the calculations. The primary path for the hydrogen abstraction from methane is via H and OH radicals. It is observed that the general behavior for more sensitive reactions was similar for the Aramco and GRI-3.0 mechanisms. With an increase in steam dilution, the sensitivity for this reaction: CH_4_ + OH ⇔ CH_3_ + H_2_O shows a decreasing trend for Aramco 3.0 mechanism and an increasing trend for the GRI-3.0 mechanism. H_2_O addition augments the reaction to follow in the reverse direction. This signifies a decline in the reactivity of the mixture with steam addition. It is to be noted that with steam dilution increasing from 0 to 30%, the chain branching reaction O_2_ + H ⇔ O + OH produces fewer OH radicals. This can be interpreted from Fig. 13c–d where the reduction in the production rate of OH radicals is observed with an increase in dilution level from 0 to 20%. Hence,OH radicals' production rate is not shown for 0–30% dilution in this section to avoid repetition. The role of HO_2_ in the H-abstraction of methane is significant and has a positive sensitivity in the Aramco mechanism. H_2_O_2_ formed from this reaction dissociates to two OH radicals which can increase the reactivity of the combustion system. With dilution, the sensitivity of this reaction reduces steadily. However, for GRI3.0, this reaction becomes insignificant. The reduction in the methane percentage alters the composition to a leaner mixture for hydrocarbon fuel only.

The production of OH radicals is immensely dependent upon the availability of H radicals. Hence, the reactions consuming H radicals like the H-abstraction of the fuel, CH_4_ + H ⇔ CH_3_ + H_2_, compete with the OH forming reactions and therefore has a large negative sensitivity. Also, this reaction is a chain propagation reaction as opposed to the chain branching reaction: O_2_ + H ⇔ O + OH, which produces two reactive radicals that can help initiate and sustain the flame. Similarly, the H producing reactions for, e.g., 2CH_3_ ⇔ H + C_2_H_5_ have a positive sensitivity. Correspondingly, the OH producing reactions such as CH_3_ + O_2_ ⇔ CH_2_O + OH, CH_2_O + O_2_ ⇔ HCO + OH, and CH_3_ + HO_2_ ⇔ CH_3_O + OH have a positive impact upon OH availability. All these reactions are again chain branching reactions. The reaction 2CH_3_ (+ M) ⇔ C_2_H_6_ (+ M), competes with the reaction between CH_3_ with O_2_ and HO_2_ to produce OH radicals and thus has a negative sensitivity.

**Figure 7:**
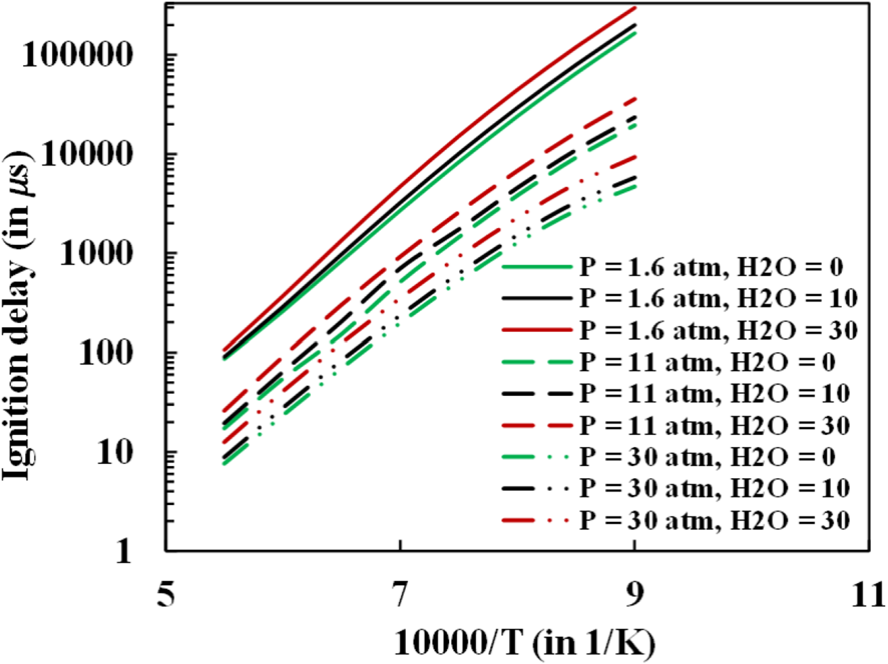
Ignition delay comparison with steam dilution (0%, 10% and 30%) and pressure (1.6, 11 and 30 atm).

The individual effect of dilution and pressure is studied using Aramco 3.0 mechanism keeping the equivalence ratio constant $(\varphi = 1)$ . Although, Gurentsov et al.^38^ have estimated ignition delay times with and without steam additionthey observed different results numerically. This makes it difficult to analyze the effect of pressure and dilution individually with their results. The steam dilution effect on the ignition delay from the numerical simulation could not be directly compared with the experimental results^29^ because of the non-uniformity of the test mixture conditions adopted in the study. The dilution level in the experimental study was also considered as a mass fraction of the fuel, which signifies that with the increase in dilution level for an equivalence ratio, the mass fraction of methane is reduced. However, since the equivalence ratio was fixed, and the mole fraction of O_2_ is a function of fuel, which is methane and steam, a direct relationship between the dilution and ignition delay was difficult to comprehend. Furthermore, the steep argon dilution (98%) for the mixture considered in the experiment is not widely used in practical applications. Therefore, ignition delay was computed with the Aramco mechanism with the mole fraction of the steam varying from 0 to 30% of the premixed mixture for temperature varying from 1111 to 1818 K and air used as the oxidizer (as shown in Fig. 7) keeping the equivalence ratio fixed at 1.

This study was carried out for the three pressure conditions adopted in the experimental study of Donohoe et al.^29^. It is observed that the diluent and pressure have a countering effect on the ignition delay. The ignition delay reduces significantly with pressure because of the contraction of the combustion zone to a narrow zone, thus enhancing the formation of the local combustion pockets. This generates a non-homogeneous combustion zone and accelerates the ignition process. Steam dilution conversely increased the ignition delay, although not in the order of the pressure effect. It was observed from the simulation that steam dilution has a minimal effect on the ignition delay characteristics, especially at high-temperature conditions. The specific heat of steam is high compared to the nitrogen (only diluent in the oxidizer for the case of no steam addition), which helps absorb more heat. This reduces the reaction temperature, which inherently reduces the system's reactivity. The steam's addition also increases the reaction rate: H + O_2_ + M ⇔ HO_2_ + M, which competes with the main chain branching reaction H + O_2_ ⇔ O + OH. HO_2_ is a more stable species than the reacting radicals like O and OH. The termolecular reaction is favored in the steam dilution case because of the high water chaperon efficiency. This can be seen from the reduced rate of H radicals consumption and OH radicals production through the reaction H + O_2_ ⇔ O + OH with steam dilution as shown in Fig. 13 in the emissions sections discussed later.

**Figure 8:**
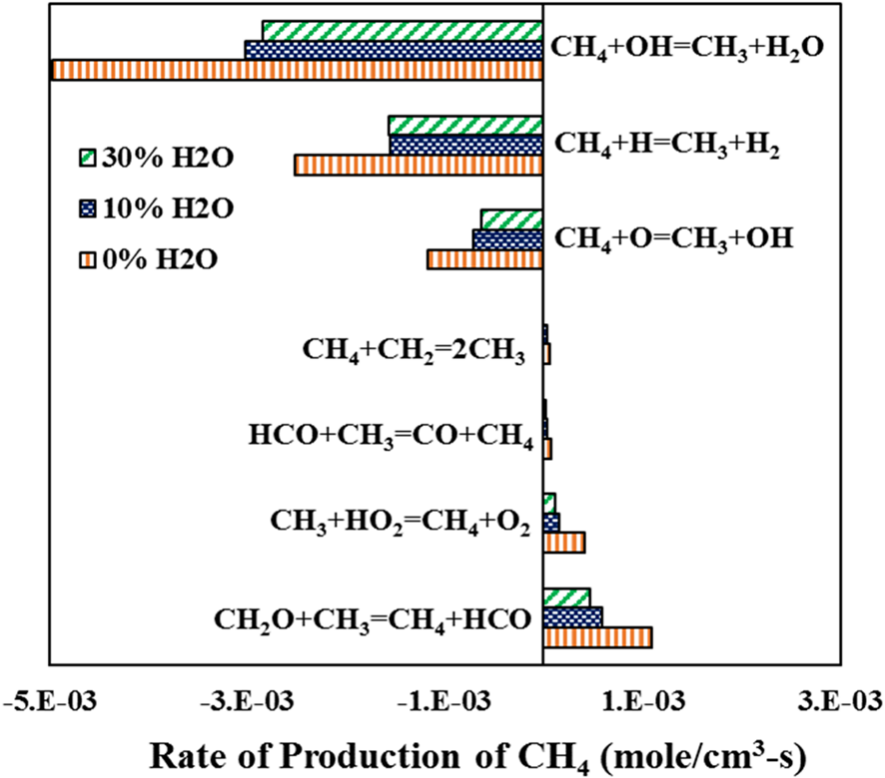
Rate of methane production for various steam dilution (0%, 10%, and 30%) conditions at 1.6 atm and 1800 K using Aramco 3.0.

The variation in the ignition delay is dependent on the decomposition rate of the fuel. Figure 8 shows the methane decomposition rate for 0, 10, and 30% H_2_O dilution at 1.6 atm pressure and 1800 K temperature. The primary reaction for methane oxidation is CH_4_ + OH ⇔ CH_3_ + H_2_O, followed by reactions with H and O radicals. The rate of decomposition of methane through these reactions reduces with steam addition. It is shown in the emissions section that the net rate of production of OH radicals reduces with the steam addition. Thus, the overall reactivity reduces, and the ignition delay increases with the dilution. However, the rate of this reduction decreases for high dilution cases. Since the computations are carried out for high-temperature conditions, the contributions from the methyl-peroxy (CH_3_OO) and methoxy (CH_3_O) radicals towards the methane oxidation are diminished rapidly compared to low-temperature combustions where these radicals play key contributors for the decomposition of CH_4_^33^. It is also observed from the work of Shareh et al.^33^ that with steam addition, the production rate of OH radicals is increased. This increment is primarily due to consideration of a very high third-body efficiency of 12 for the reaction: H_2_O_2_ (+ M) ⇔ 2OH (+ M), compared to moderately high third-body efficiencies of 6–8 for H_2_O in the mechanisms considered in the present study. The low third-body efficiency and the different temperature regimes resulted in the decrease in the OH production with the dilution. A contradiction is observed for the recombination reactions where the methane is formed. The steam dilution reduces the rate of formation of these recombination reactions which could reduce the ignition delay. However, since the relative contributions from these reactions have very small magnitudes towards producing CH_4_, the overall effect of the dilution is towards increasing the ignition delay. At higher pressure, the collision efficiency increases and there is a greater chance of ignition as well as sustaining of the ignition. Similarly with rise in temperature, extra thermal energy is supplied which helps reactions to occur by overcoming the thermal barrier. The temperature and pressure effect in steam diluted environments behaves similarly to the undiluted case.

### Laminar flame speed

**Figure 9:**
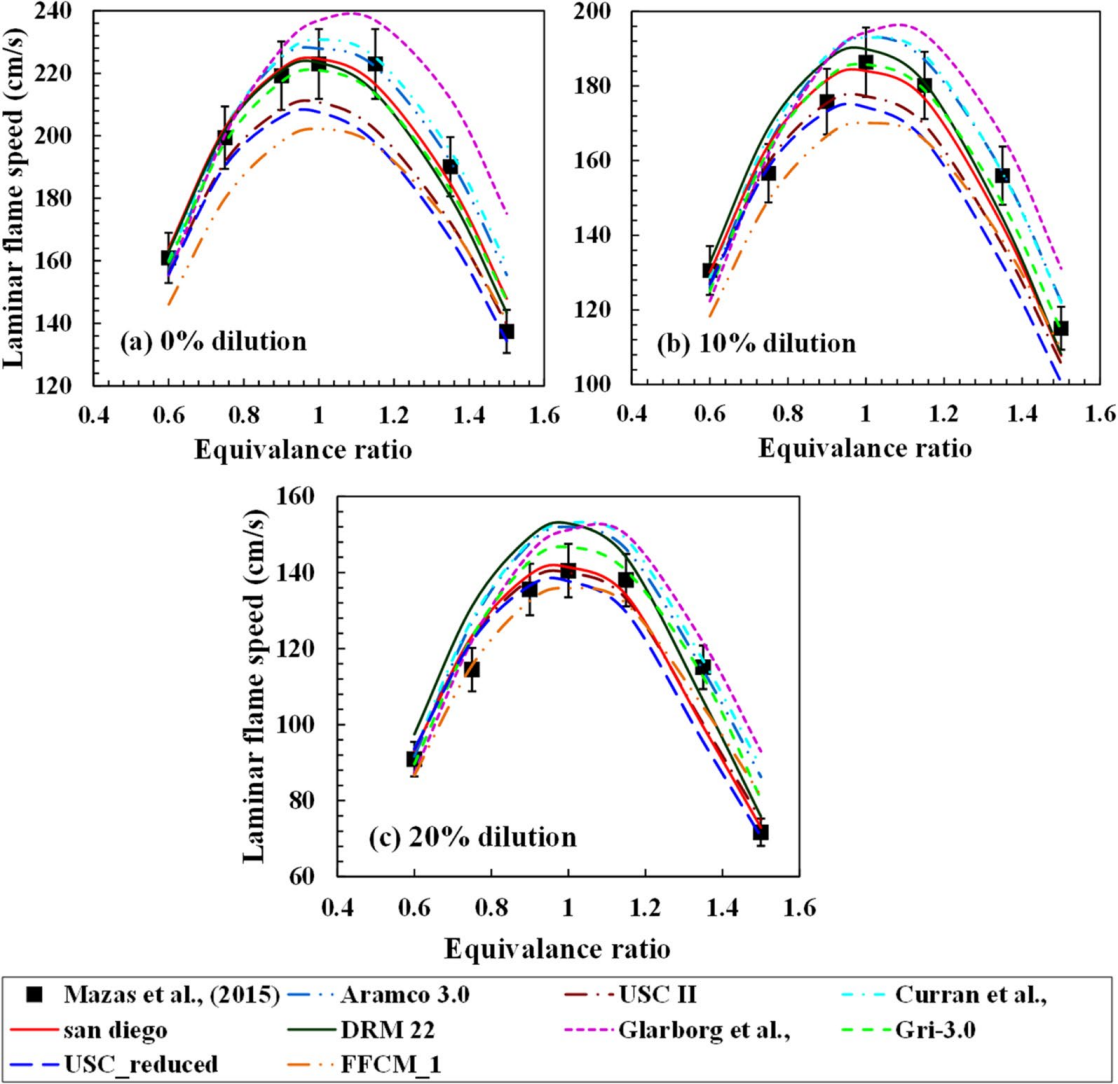
Laminar flame speed for CH_4_/O_2_/N_2_/H_2_O mixture at an unburned gas temperature of 373 K and atmospheric pressure with oxygen enrichment of 0.5 for (**a**) 0% (**b**) 10% and (**c**) 20% steam dilution.

The experimental estimation of laminar flame speed variation with steam dilution from the works of Mazas et al.^37^ and Galmiche et al.^34^ are considered in the present study. Mazas et al.^37^ have considered premixed combustion with oxygen-enriched and steam dilution environments for methane. In the present study, the oxygen enrichment ratio of 0.5 (equal moles of oxygen and nitrogen in the oxidizer) is considered, and the dilution is varied from 0 to 20%, as shown in Fig. 8. The equivalence ratio is varied from 0.5 to 1.5. The flame speed reduces gradually for all equivalence ratios with steam dilution. It was observed that at the lean mixture conditions, each mechanism has a good match with the experimental results and converges with each other. However, for the rich mixture conditions, slight deviations are observed between the computed results from different mechanisms. The maximum difference between the computed results from the different mechanisms at the richest conditions $(\varphi = 1.5)$ is more than twice at the leanest condition $\varphi = 0.5$. Mazas et al.^37^ have considered an uncertainty of 5% for their laminar flame speed estimation,which is shown in Fig. 9 correspondingly.

Figure 11a shows the cumulative relative error for all equivalence ratios at 0%, 10% and 20% dilution. Glarborg mechanism over predicts for all dilution levels, although the deviation reduces with dilution. It is also observed that the peak flame speed computed with Glarborg model is slightly at a richer mixture as compared to the rest of all mechanisms. The FFCM-1 model shows a large deviation in estimating the laminar flame speed for lean mixtures at no dilution case as compared to 10% and 30% dilution cases. USC II and its reduced model under predicts at 0% steam. However, it has a good match at 30% steam. The error incurred during the computation reduces steadily with dilution. A similar observation was found for the FFCM-1 model. San Diego mechanism gives the best estimate for all dilution cases, with the deviation increasing marginally with dilution. GRI-3.0 and DRM-22 also show a fair match with the experiments at no and intermediate dilution, but the errors at 20% dilution are observed to be high. Similar behavior is seen for the Curran mechanism, where the estimation deviates largely at the high dilution case.

**Figure 10:**
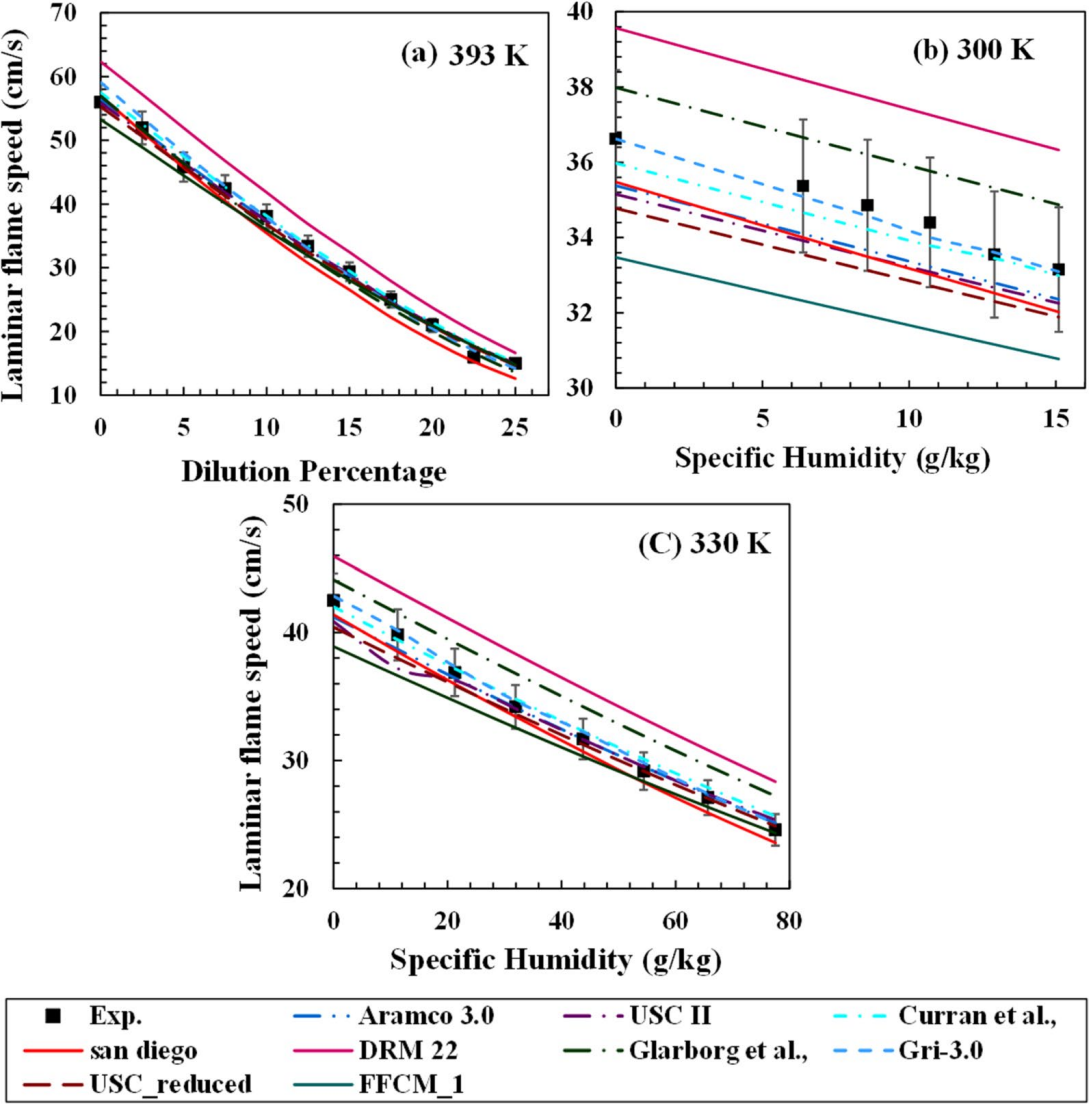
Comparison of different mechanisms with dilution varying (**a**) from 0 to 25% steam in CH_4_/air mixture at 393 K,^34^ and (**b**,**c**) from 0 to 100% relative humidity in CH_4_/air mixture at 300 K and 330 K.^40^.

Galmiche et al.^34^ conducted experiments with different diluents like nitrogen, carbon dioxide, steam, a mixture of these species, argon, and helium to study the effect of dilution on the laminar flame speed at 1 atm and 393 K for methane/air flames. The dilution levels were varied from 0 to 25% on a molar basis, and the equivalence ratio of 1 is maintained constant across all data points. This study has a high importance because of the use of the air as the oxidizer which helps in analyzing the combustion chemistry in practical scenarios accurately. Controlling nitrogen and oxygen separately and supplying them to the combustion domain in different ratios is complex and not practical in many cases. In this study, the steam is added to the premixed mixture and the mole fractions of methane, nitrogen, and oxygen are varied accordingly to maintain the stoichiometric mixture. As observed in the previous set of experiments, the flame speed reduces continuously with the increase in dilution. All mechanisms were run for the above operating conditions and are shown in Fig. 10a. DRM-22 mechanism over-predicts the flame speed slightly more than the other mechanisms for all the dilution cases. San Diego mechanism estimated laminar flame speed fairly well at the low dilution range. However, the deviations slightly increased for the high dilution cases. The USC II, Aramco 3.0, Curran et al. mechanism, USC_reduced, GRI-3.0, Glarborg et al., and FFCM-1 mechanism could predict flame speed precisely in the decreasing order, with USC II being the most accurate mechanism. An uncertainty value of 5% associated with the experimental estimation is shown in Fig. 10a.

**Figure 11:**
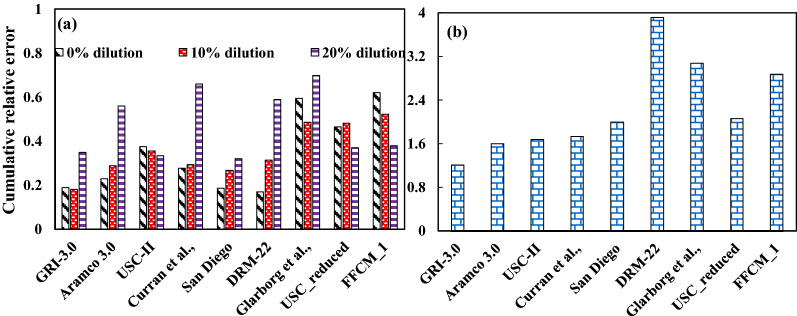
Cumulative relative error during the prediction of laminar flame speed (**a**) to show the effect of steam addition on error propagation using cases for Mazas et al.^37^ and (**b**) for combined steam dilution cases adopted in literature^34,37,40^.

Figure 10b,c show the comparison of laminar flame speed computation using mechanisms considered from the present study with the experimental results of Boushaki et al.^40^. Steam is added as specific humidity in the air up to 100% relative humidity. The experiments were carried out at atmospheric pressure and two temperature cases of 300 K (Fig. 10b) and 330 K (Fig. 10c), with an error percentage of 5% associated with the experimental measurements. The GRI-3.0 mechanism was able to predict the best of all mechanisms for the two temperature conditions, followed by Curran’s and Aramco-3.0 mechanisms. FFCM-1 under-predicts laminar flame speed significantly, whereas DRM-22 over-predicts by a large degree. The CRE index for the laminar flame speed considering all datasets from the literature^34,37,40^ is shown in Fig. 11b. GRI-3.0 mechanism has the least CRE index from the current dataset with Aramco-3.0, Curran, and USC II mechanism also predicting the experimental results significantly well. Since all the cases considered in the present study are at atmospheric pressure conditions, the detailed mechanisms have low errors compared to the ignition delay estimation, where cases with high high-pressure conditions were also adopted. The errors in the reduced mechanisms and Glarborg mechanism are considerably large compared to the rest.

**Figure 12:**
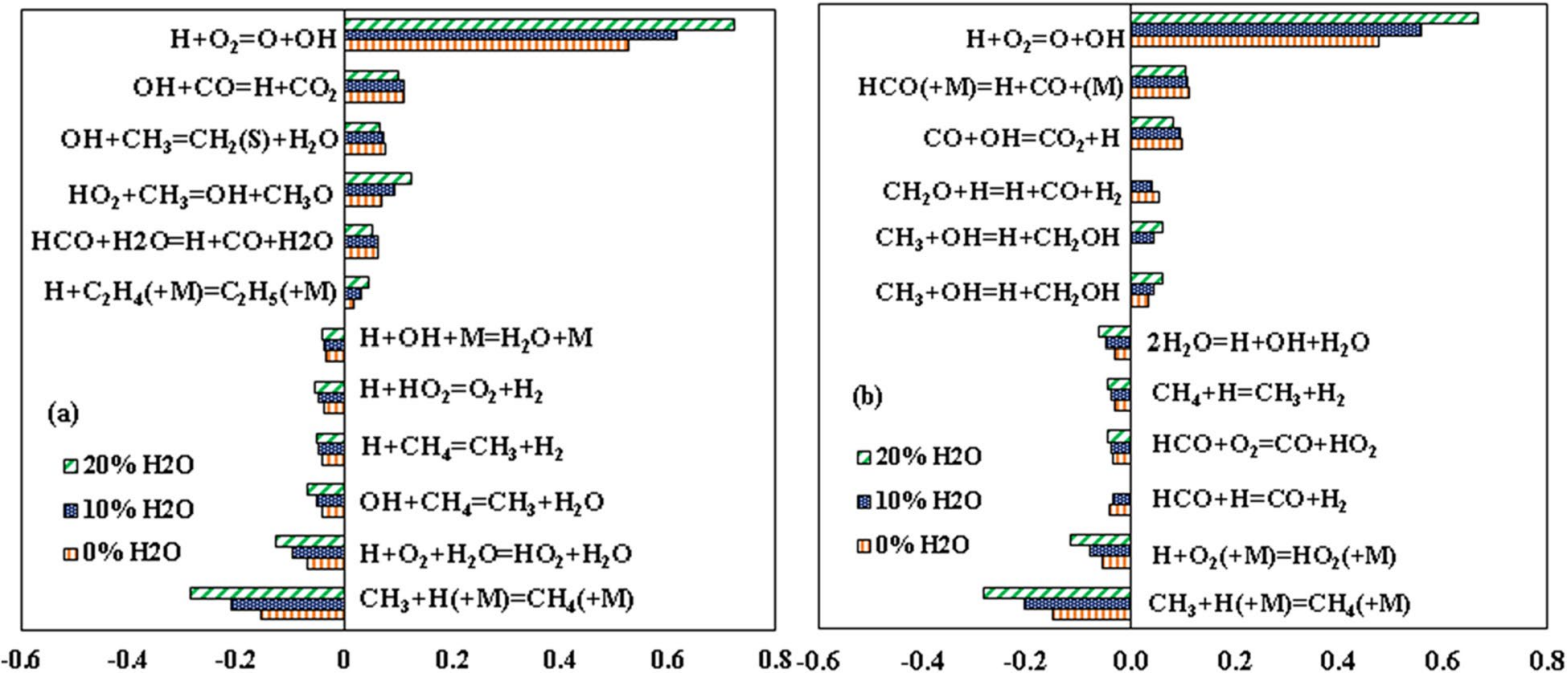
Normalized sensitivity coefficient for the flow rate using (**a**) GRI-3.0 and (**b**) Glarborg mechanism for 0%, 10% and 20% dilution^34^.

The sensitivity study for the laminar flame speed is carried out to identify the important reactions and pathways with different dilution levels. This sensitivity analysis is generally carried out by perturbing the flow rate of the individual reaction rate by a small value and monitoring the effect of this change on the laminar flame speed. The laminar flame speed is proportional to the flow rate, and thus analyzing flow rate will help understand the reactions affecting the laminar flame speed at high dilution levels. A similar procedure has been adopted by many researchers in the literature to do sensitivity analysis for laminar flame speed^33,52,56^. The experimental operating conditions of Galmiche et al.^34^ are considered for this study. GRI-3.0 mechanism is adopted for the sensitivity study as it has the least CRE index. Glarborg mechanism performed unsatisfactorily in computing laminar flame speed for all experimental conditions and therefore adopted for the sensitivity study to understand the inconsistency between the mechanisms. The sensitivity analysis for 0%, 10% and 20% steam dilution is shown in Fig. 12a,b.

The major 12 reactions that have the most influence on the flame speed are considered in this study. The chain branching reaction H + O_2_ ⇔ O + OH has the highest positive sensitivity. Similarly, the chain-terminating reaction H + OH (+ M) ⇔ H_2_O (+ M) and CH_3_ + H (+ M) ⇔ CH_4_ (+ M) has a high negative sensitivity coefficient. All the major reactions with high normalized sensitivity coefficients have an increased affinity at high dilution cases. The H and OH radical removing reactions from the reacting zone have a very negative effect on the propagation and sustenance of the flame. Although the behavior of these high-sensitivity reactions is similar for the two mechanisms, the reactions with moderate sensitivities differ significantly. The H-abstraction of methane from OH radicals, an important reaction for the chain initiation step, is less important for the Glarborg mechanism. This reaction has a negative sensitivity towards the flow rate, and its sensitivity increases with dilution, as can be observed from the GRI-3.0 mechanism. The over-prediction of the Glarborg mechanism, as seen from Fig. 8, may be due to the reduced influence of the negative sensitive reactions.

### Emissions

**Figure 13:**
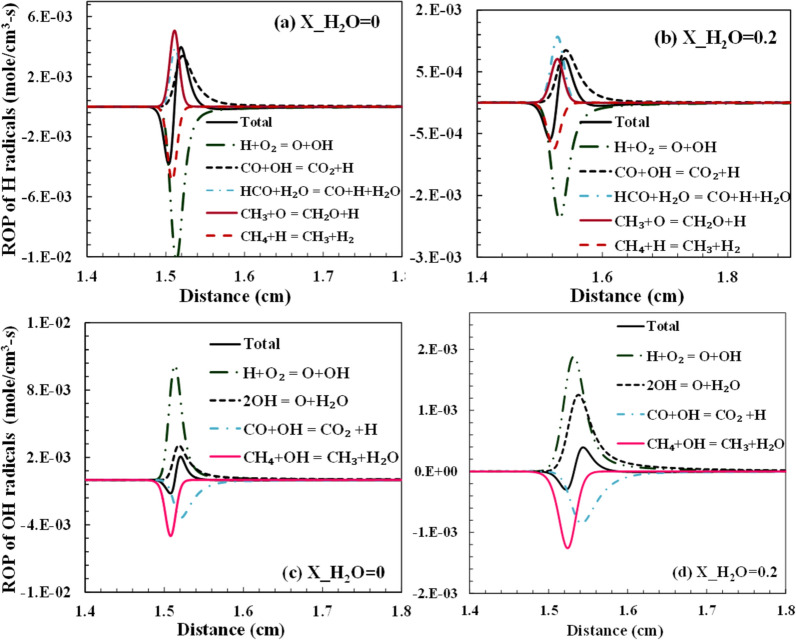
The net rate of production of (**a**–**b**) H and (**c**–**d**) OH radicals with 0% and 20% steam dilution using Aramco-3.0 mechanism.

The two types of emissions analyzed in this present study are CO and NO. All hydrocarbon combustion produces CO as a pollutant, and the net rate of CO formation increases with a decrease in temperature leading to incomplete combustion. NO is either formed from the nitrogen in the oxidizer or from the fuel-bound nitrogen. Since no fuel-bound nitrogen is considered in the computations, NO is formed from the reactions of atmospheric N_2,_ which is essentially an inert gas. The NO is majorly thus produced as thermal NO_x_. The thermal NO formation is usually determined from the Zeldovich mechanism, which suggests that O and OH radicals are important for the oxidation of the nascent N radicals. The reaction temperature also reduces due to the dilution, which signifies the importance of prompt NO_x_ in these conditions. From the sensitivity analysis performed for the OH and the flow rate in the previous sub-sections, it was evident that the H and OH radicals significantly affect the kinetics of the combustion. Steam dilution directly affects the concentration of these radicals because of the dissociation of H_2_O species. Considering the reactions: 2OH ⇔ O + H_2_O and H_2_O (+ M) ⇔ H + OH (+ M), it shows the role of H_2_O in driving the reaction kinetics. The pollutants, namely CO and NO, are formed and dissociated with the help of these radicals, which affects their net rate of production. The experimental operating condition adopted in Galmiche et al.^34^ is used to analyze the radicals' formation rate and emissions. The total net rate of production and consumption of H and OH radicals are shown in Fig. 13. It is observed that the primary chain branching reaction determines the consumption of H and production of OH radicals largely. The steam dilution has a reducing effect on the net rate of these radicals in the reaction zone. This reduces the overall reactivity of the system, which increases the ignition time. The change in the H radicals advances to a downstream location for the steam dilution case, as shown in Fig. 13a,b. The change in these radicals is observed to be very steep for the case of no dilution compared to the dilution case, which denotes that a wider reaction zone is formed with steam dilution. In the mechanisms considered for this study, nitrogen chemistry isn’t available in each of them. Only Glarborg et al., and GRI-3.0 mechanism have the NO_x_ chemistry available. Although both mechanisms agree with the experimental results for laminar flame speed and ignition delay, for the experimental results of Galmiche et al.^34^, which uses air as the oxidizer, GRI-3.0 performs marginally better and thus is selected for the NO formation analysis with dilution.

**Figure 14:**
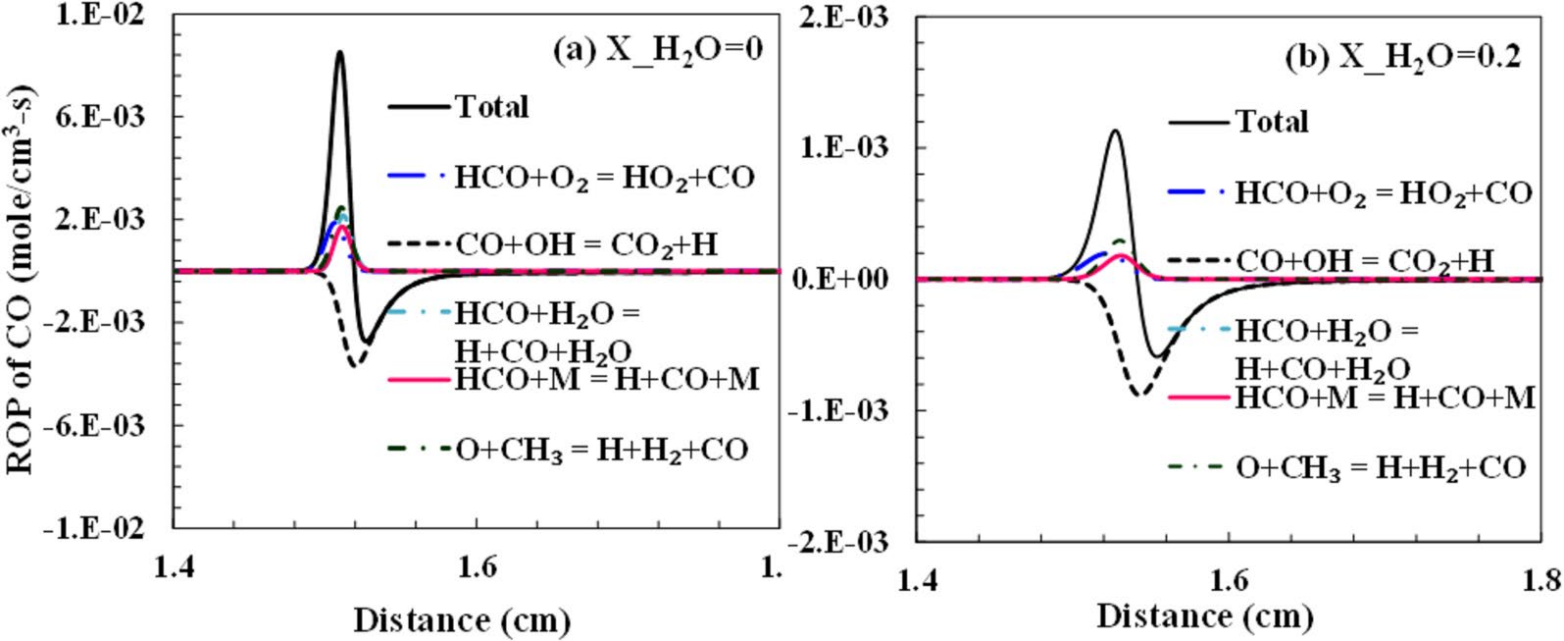
Net rate of production of CO with (**a**) 0% and (**b**) 20% steam dilution using Aramco mechanism.

**Figure 15:**
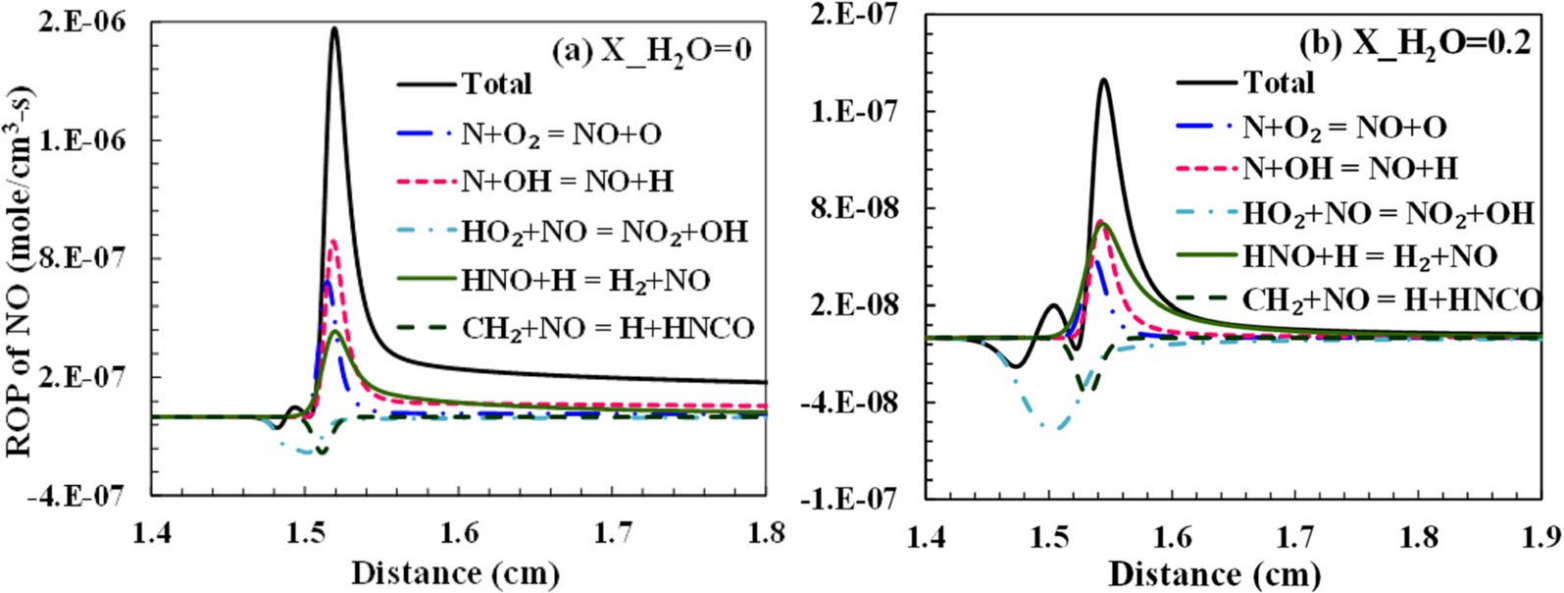
The net rate of NO production with (**a**) 0% and (**b**) 20% steam dilution using the GRI-3.0 mechanism.

Figures 14 and 15 show the net production rate of CO and NO, respectively, through the major reactions. The total CO formation rate reduces significantly from 8E-03 to 1E-03, with steam dilution increasing from 0 to 20%. The exothermic reaction CO + OH ⇔ CO_2_ + H has the most negative rate of formation, signifying the oxidation of CO to CO_2_ majorly and has the biggest impact on the net formation rate of CO. The total rate of production of CO as well as from all contributing reactions reduces significantly with steam dilution. The sharpness of CO and NO emissions peaks reduces with the dilution similar to that of the H and OH radicals. This suggests that a more distributive reaction region is achieved with dilution. Thus, the oxidation of CO takes place over a wider zone under the dilution case. A similar sharp reduction is observed in NO with steam dilution (Fig. 15b). The reduction of NO formation is primarily via the thermal process. The steam dilution reduces the overall temperature of the combustion zone owing to its high specific heat. This reduces the minimum thermal barrier needed to form the thermal NO through the Zeldovich mechanism. The prompt NO has a small role in NO's total net production rate.

### Reaction pathway

**Figure 16:**
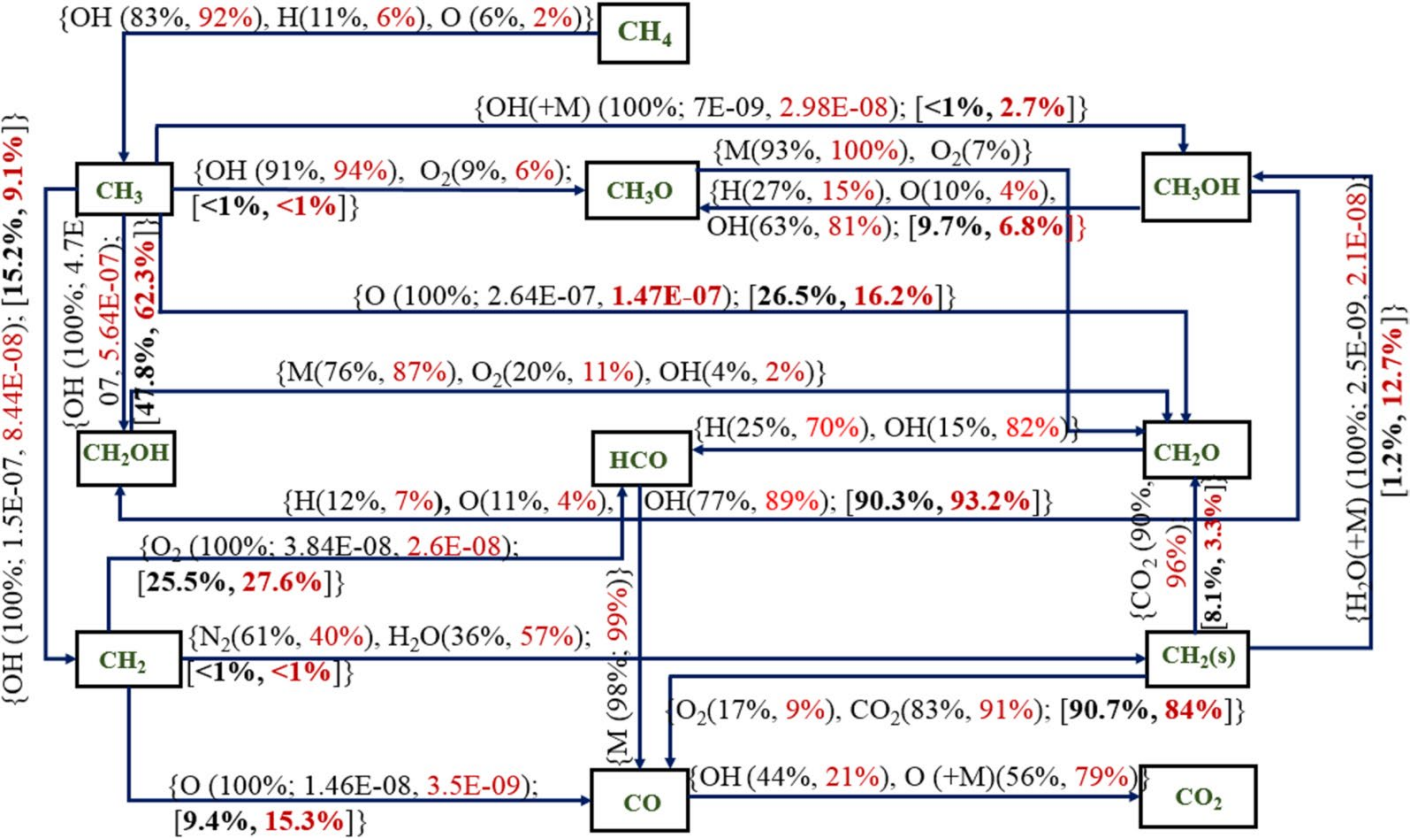
Methane–air combustion with no dilution (values in black color) and 20% dilution (values in red color) at 393 K and *P* = 1 atm for Aramco 3.0 mechanism with a residence time of 0.5 s, and the values within the square bracket represents the branching percentage of the particular reaction from the precursor intermediate species.

**Figure 17:**
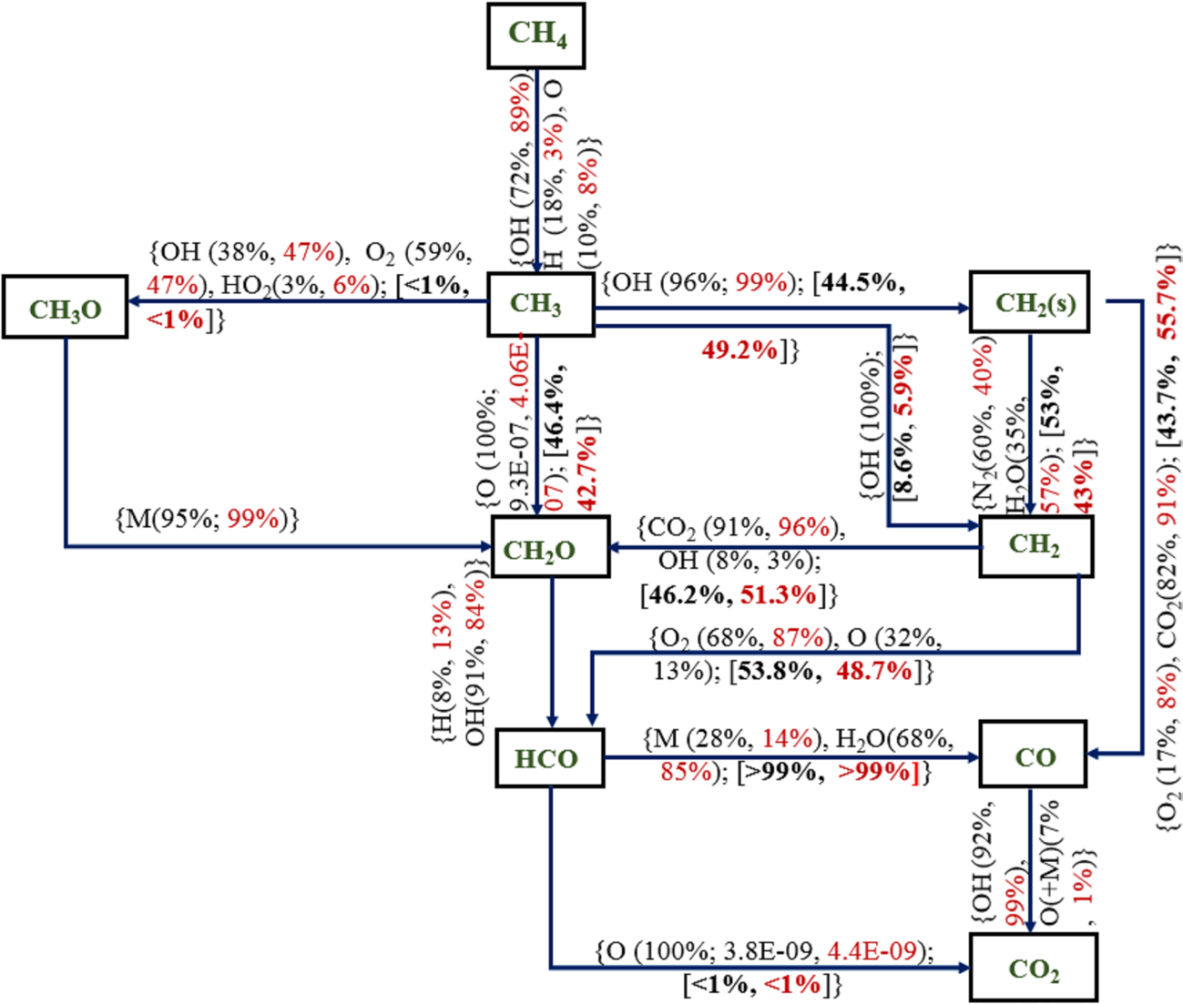
Methane–air combustion with no dilution (values in black color) and 20% dilution (values in red color) at 393 K and *P* = 1 atm for DRM-22 mechanism with a residence time of 0.5 s and the values within the square bracket represents the branching percentage of the particular reaction from the precursor intermediate species.

In this section, a detailed and a reduced mechanism are considered to study the oxidation of methane to its final equilibrium product (CO_2_) for the compositions used in the work of Galmiche et al.^34^ and investigate the effect of the number of species and reactions on the key combustion parameters. All detailed mechanisms showed similar pathways, however, with different rates of reaction. For detailed mechanism, Aramco 3.0 was chosen as it showed good prediction under oxy/air combustion cases for no dilution as well as with dilution conditions, and DRM-22 was chosen as the reduced mechanism as this has the least number of species and predicted parameters with large errors as compared to other mechanisms. The overall reaction pathway for the methane combustion under 0 and 20% dilution using Aramco-2.0 and DRM-22 mechanisms are shown in Figs. 16 and 17, respectively. For this, a perfectly stirred reactor model is adopted to analyze the pathway with the reactor temperature, and the pressure is set to 1300 K and atmospheric condition, respectively, for a constant residence time of 0.5 s. Figure 16 shows the reactions for conversion between two species. The relative formation from each reaction between them is shown in black for zero dilution and red for 20% steam dilution condition. For the reactions contributing 100% for the two species considered, the net rate of formation of the leading species is shown for the no dilution and 20% dilution cases. The branching ratios of the intermediate species like CH_3_, CH_2_, and CH_3_OH species through different routes are given in percentage terms (shown in square brackets) to show the predominant pathway for the oxidization of methane. CH_4_ primarily undergoes the H-abstraction process through reacting with the OH to produce the methyl radical. This route becomes even more significant with steam dilution than the H-abstraction through H and O radicals. Methyl radical oxidizes further with the OH radical to form hydroxymethyl radical, the predominant path in the Aramco 3.0 mechanism. With steam dilution, the CH_3_–CH_2_OH pathway becomes more dominant than the zero dilution case. CH_2_OH is also formed after removing hydrogen from the methanol via reacting with H, O, and OH radicals. CH_2_O is produced from the reaction between the hydroxymethyl radical and, majorly, a third-body species. This reaction path is enhanced with the steam dilution owing to its high large third-body efficiency. CH_2_ radical further reacts with the hydroxyl radical to form CH (not shown in the reaction pathway), which reacts to form CO through oxygen molecule or by first oxidizing to C radicals.

In the DRM-22 mechanism, the CH_2_O radicals are directly formed from methyl oxidation. From the pathway diagrams, it is observed that the bicarbonate (HCO) ion is a pivotal species for producing CO species. The sensitivity of this reaction is also high for the formation rate of CO. In Aramco 3.0 mechanism, this oxidation is through collision with a third-body species only irrespective of dilution levels. However, in DRM-22, another route for this formation is observed through H_2_O. Thus, steam dilution has a direct influence on the CO formation rate. The second route for CO formation is through methylene radicals reacting with the O radicals. The reaction between CH_2_–CH_2_ (s) is observed to be in the opposite direction for Aramco 30 and DRM-22 mechanisms. This reaction is, however, dominant for the DRM-22 mechanism only. The oxidation of CO–CO_2_ is primarily through reacting with the OH radical and O radicals in the presence of a third-body species. The influence of steam as the third-body species is again found to be dominating in the oxidation of CO–CO_2,_ as seen from the higher percentage of the third-body reaction for the higher dilution condition for Aramco-3.0 mechanism. However, this behavior is not reflected in the DRM-22 mechanism, where the OH route is observed to be dominant.

## Conclusions

In the present study, nine mechanisms are considered to study methane combustion under steam dilution conditions. The mechanisms' performance is compared by computing the experimental results from the literature using the CRE index. The chemical kinetic analysis, rate of emissions formation, and reaction pathway analysis in the diluted steam conditions are investigated. The following observations were noted.

1. For low operating pressure conditions, the GRI-3.0 mechanism gives an excellent prediction. The computations from GRI-3.0 showed the least CRE index for laminar flame speed predictions. The sensitivity analysis shows that the moderately negative sensitive reactions were not important for the Glarborg mechanism that has poorer prediction (over-prediction) for the flame speed as opposed to the USC II mechanism, which gives a better prediction.
2. For the ignition delay computations of methane under the steam dilution, the Aramco-3.0 mechanism and the Curran et al.^49^ were able to predict most closely the experimentally observed values. Since higher pressure conditions are considered in these cases, it can be suggested that while computing for applications like gas turbines and furnaces, these mechanisms can be adopted to give good results. However, since both mechanisms are detailed mechanisms, the computational cost may increase. San Diego mechanism can be chosen for low computational facility purposes as it shows very good predictions for both ignition delay and laminar flame speed computations.
3. Sensitivity analysis is carried out for these conditions with the fit mechanism to identify the key reactions and pathways. Sensitivity study for the OH concentrations shows that the H-abstraction of methane from OH radicals has an opposing trend with dilution for Aramco and GRI-3.0 mechanism. A greater error was detected during the predictions from the reduced mechanism compared to the detailed mechanisms.
4. The effect of steam dilution on the CO and NO emissions are studied using the GRI-3.0 mechanism, and a sharp reduction in the total net rate of production was seen with dilution increased from 0 to 20%. The role of OH radicals was observed to be crucial in the oxidation of CO to CO_2_.
5. No significant difference in the reaction pathway was noted with the dilution increased from 0 to 20%. Steam as a third-body species has an important effect on the oxidation of the fuel to CO_2_ for the Aramco-3.0 mechanism, while for the DRM-22 mechanism, it is observed that the effect of steam on the OH radicals has a bigger impact.

However, it is observed that there isn’t a comprehensive range of experimental data available for steam diluted methane combustion in literature, as compared to only methane combustion, which is necessary for a better assessment of the mechanisms as compared to C1–C4 hydrocarbons without diluents. This warrants further experiments to provide the extensive data needed.

## Abbreviations

CRE: Cumulative relative error
CFD: Computational fluid dynamics
ROP: Rate of production
